# Pharmaceutical system strengthening in Uganda: implementing a holistic, evidence-informed, long-term strategy

**DOI:** 10.1186/s40545-018-0150-1

**Published:** 2018-10-15

**Authors:** Martin Oteba, Anita Katharina Wagner, Morries Seru, Martha Embrey, Birna Trap

**Affiliations:** 1 Management Sciences for Health, Plot 15, Princess Anne Drive, Bugolobi, P.O. Box 71419, Kampala, Uganda; 2 Harvard Pilgrim Health Care Institute, 133 Brookline Avenue, 6th Floor, Boston, MA 02215 USA; 3 Ministry of Health, Pharmacy Department, Lourdel Road, Wandegeya, Kampala, Uganda; 4 Management Sciences for Health, 4301 N. Fairfax Drive, Suite 400, Arlington, VA 22203 USA

**Keywords:** Pharmaceutical system strengthening, Pharmaceutical sector development, Logistic system development, Supply chain

## Abstract

A strong pharmaceutical sector is a precondition for effective and efficient health care and financing systems, and thus for achieving the best possible health of a population. Supported by visionary, long-term donor funds, in conjunction with mutual trust, the USAID-funded Securing Ugandans Rights to Essential Medicines (SURE) and Uganda Health Supply Chain (UHSC) program engaged in a close, more than 10 year-long (in 2018) collaboration with the Ministry of Health of Uganda. Over time, the partnership implemented numerous multi-pronged comprehensive changes in the pharmaceutical sector and conducted research to document successes and failures. We describe the evolution and key characteristics of the SURE/UHSC interventions.

## Background

This paper introduces the *Journal of Pharmaceutical Policy and Practice* theme series on medicines management in Uganda. Together, the series publications to date [1, 6] summarize the results of the implementation and evaluation of a long-term, visionary pharmaceutical system development strategy in Uganda. The strategy was led by the Ministry of Health’s (MOH) Pharmacy Department in collaboration with two US Agency for International Development (USAID) supported programs—Securing Ugandans Rights to Essential Medicines (SURE) (2009–2014) [7] and its successor, Uganda Health Supply Chain (UHSC) (2014–2019). Here, unique aspects of SURE/UHSC are highlighted, system strengthening interventions are documented, including lessons learned, and a vision for the future is shared.

## What is unique about the health system strengthening program in Uganda?

The Uganda SURE/UHSC programs designed a comprehensive set of interconnected interventions to strengthen the country’s pharmaceutical sector. The planned interventions were piloted and evaluated to understand what works and what does not and changes were integrated into the country’s health system.

**Fig. 1:**
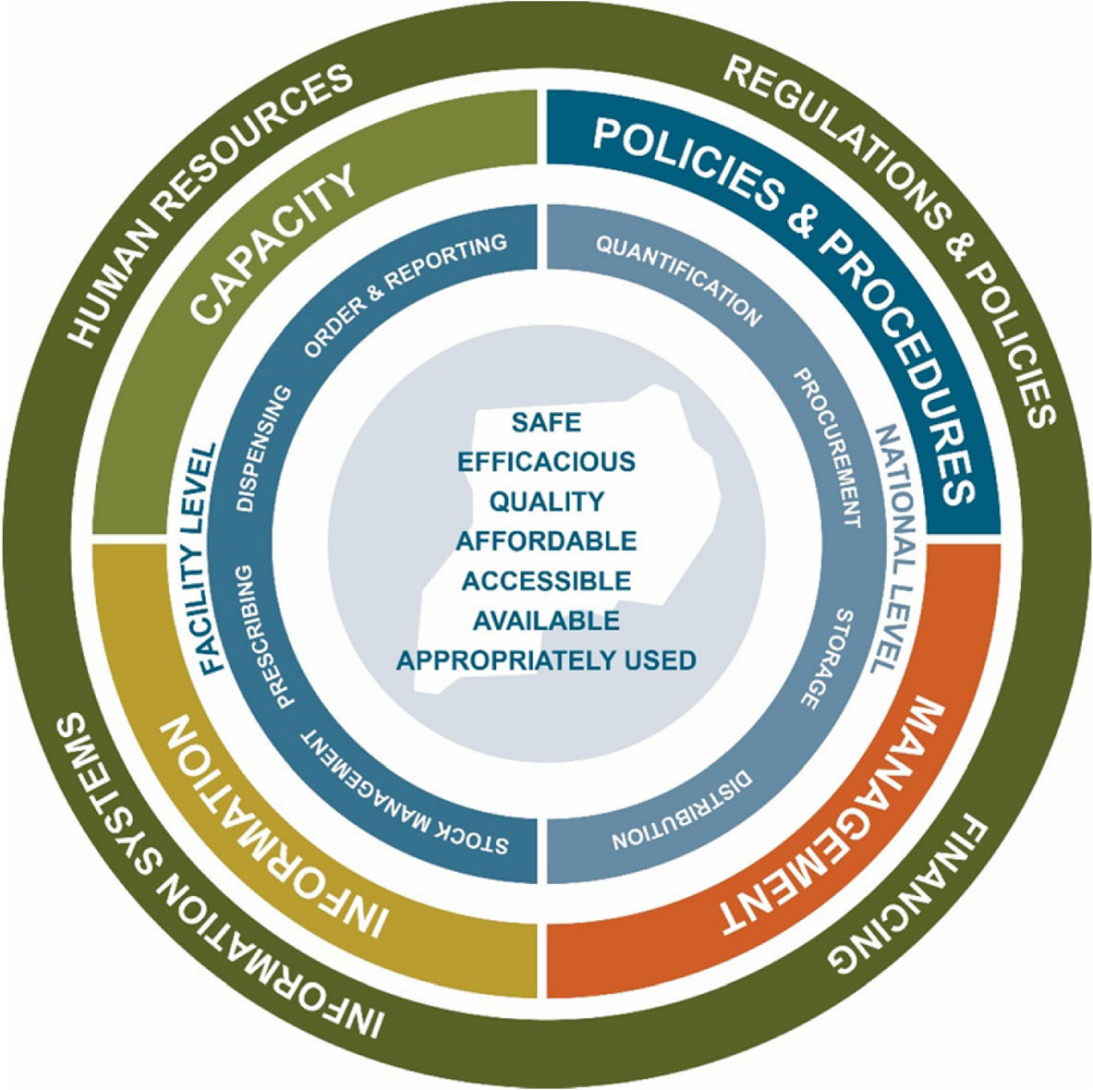
Action-oriented pharmaceutical sector strengthening cycle depicting priority action areas, levels, and goals for Uganda

An options analysis in 2010 conducted jointly by the MOH and USAID [8] identified long-standing suboptimal areas in Uganda’s essential medicines supply chain. In response, USAID funded the SURE and UHSC programs to develop and implement a comprehensive and sustainable intervention strategy to improve medicines management. The strategy is based on the understanding that a supply chain cannot function without adequate human resources, information systems, financing, and evidence-informed regulations and policies, and that interventions need to consider the vertical and horizontal interconnectivity between these elements (Fig. 1).

SURE/UHSC interventions focus on strengthening existing human resource capacity, building lasting information systems that also generate routine monitoring & evaluation (M&E) data, managing medicines and finances, and supporting national health policy development to ensure that new interventions are anchored in the system through routine procedures and practices.

*Human resource capacity* was strengthened by improving the skills of individual health facility staff members in medicines management including stock and storage management, ordering and reporting, and rational drug use and by building the capacity of training institutions to teach medicines management. Initially, health staff responsible for pharmaceuticals in about half (*n* = 1499) of all government and private not-for-profit (PNFP) health facilities covering about half of all the districts were targeted for in-service supervision by trained medicines management supervisors (MMS) [1]. A new multipronged approach, Supportive Supervision, Performance Assessment, and Recognition Strategy (SPARS), was implemented by training existing local district government and PNFP staff members as MMS [1].

Makerere University was tasked with institutionalizing the training and testing of the MMS. The MMS take personal responsibility, are accountable, and receive incentives for implementing SPARS under the leadership of the district health officer.

MMS motivate and support front-line health workers to improve how they manage medicines along all supply chain functions [1]. The health workers as well as the MMS are encouraged and motivated by the performance assessment carried out during each supervisory visit and which is linked to a recognition scheme tailored to both the health worker and the MMS.

*A robust and sustainable information system* is needed to identify problems, assess the impact of pilot interventions, and routinely monitor and evaluate system performance.

SURE developed the Pharmaceutical Information Portal (PIP) in 2013, now residing in the MOH, as a database for SPARS facility performance data and other sector-related data to be used by managers at all levels to manage pharmaceuticals and facility performance [1]. Existing pharmaceutical system performance measures were used or new ones developed as appropriate to design the SPARS performance measures. Government staff can query PIP in real time about medicines management performance in each of about 3000 government and PNFP health care facilities and they can aggregate information across facility, district, regional, and national levels. For example, a recent PIP query revealed a serious problem with suspected malaria cases that tested negative, yet received artemisinin-based combination therapy. As a result, policy makers restricted the use of such medicines to cases that test positive and instituted the use of rapid diagnostic tests at all levels of care.

The financing skills of individual health facility staff members in government and PNFP facilities were strengthened by providing supervision in budgeting and expenditure management for health commodities, by developing standard operating procedures, and by building the capacity of training institutions to teach pharmaceutical and health commodity financial management as part of their pre-service curricula.

A new financial and commodity tracking system (FACTS) was designed to be implemented at national medical stores and central ministerial levels. The existing supply chain systems were harmonized, standardized and optimized to more accurately quantify needs, and introduce order and delivery schedules, and a national quantification and procurement planning unit (QPPU) was established. The supply system was also streamlined from national stores to facility level by introducing the concept of “one supplier - one facility” and by rationalizing supply management at facility level to have only one stock card for each item per facility.

To sustain successful interventions and ensure they could be adapted to changing needs, interventions needed to be integrated into existing *policies, regulations, procedures, and practices*. Although, like other donor-driven programs, much SURE/UHSC support takes place at the district level, however, SURE/UHSC was designed from the beginning to connect closely to the central-level MOH, through strategies including staff secondments to the Pharmacy Department, Planning Department, and several priority disease programs. This assured that from the start, the MOH would own the interventions and transform them into national policies and practices. Examples include the following:

- SPARS was made a national strategy, rolled out nationwide, and expanded from focusing on essential medicines to include antituberculosis and antiretroviral treatment, laboratory services, and pharmaceutical financial management;
- RxSolution, an electronic logistics management information tool, was piloted and chosen for rollout at all higher-level facilities [7];
- the inspection of public sector facilities for Good Pharmacy Practices (GPP) became a legal requirement that links to SPARS performance to GPP accreditation;
- the National Medicines Policy and pharmaceutical sector M&E system were updated to integrate the new initiatives and;
- the curricula to train health workers such as pharmacists and pharmacy technicians, nurses and nurse aids, laboratory technicians, physicians, and clinical officers, were revised to include training in medicines management components (stock and storage management, ordering and reporting, rational medicines use); moreover, the MMS training components including supportive supervision, RxSolution, and pharmaceutical financial management became part of the curriculum for pharmacy students at Makerere University.

The Ministry of Health, SURE/UHSC program leadership, and USAID have been committed to designing change, piloting change, assessing impact of change, and scaling up change if the interventions were documented as feasible and effective. In those cases, the Ministry of Health translated the successful interventions into new policies that were implemented nationwide and by all donors. Interventions were rigorously evaluated to share lessons learned and avoid replication of unsuccessful approaches [9]. Health system research played an important part of the planning and the funding. Following eight years of program implementation, program staff conducted more than 20 evaluations of new interventions, which were designed and carried out through close collaboration between program staff, MOH and district staff, and with assistance from academic colleagues at Makerere University and Harvard Medical School [1–6, 10, 11]. The continuous evaluation approach has prompted additional changes; for example, operational research revealed that MMS training needed more focus on supportive supervision [10]. Furthermore, an evidence-focused mindset has been instilled among MOH staff at all levels and has supported the professional development of several program and MOH staff members and future managers.

Interventions to strengthen pharmaceutical sector in Uganda are summarized in Table 1.

**Table 1:**
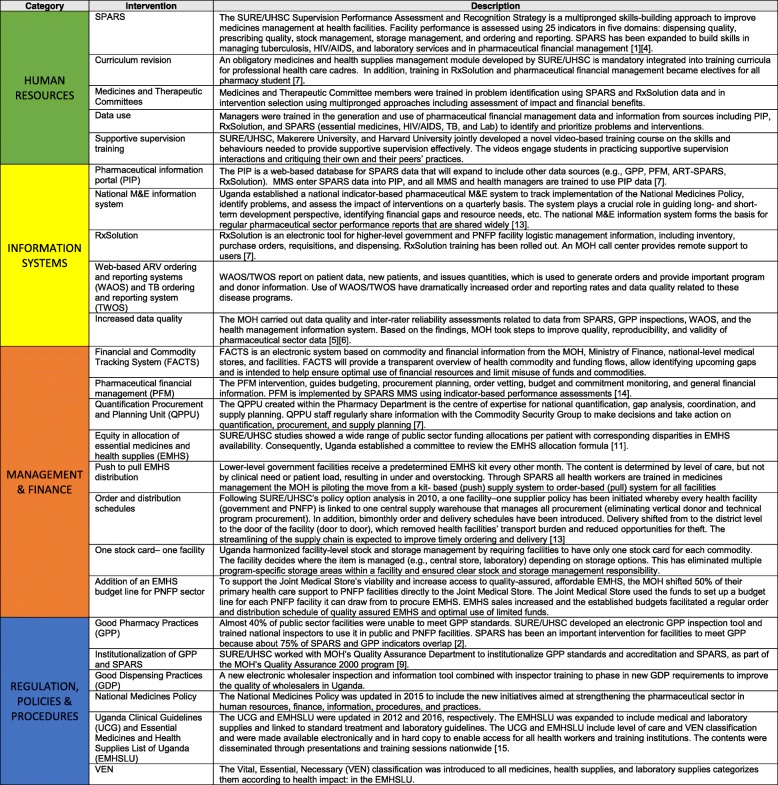
Interventions to strengthen the pharmaceutical sector in Uganda

## What made this unique program happen?

A confluence of several factors enabled the SURE/UHSC program. The HIV/AIDS pandemic prompted large donor investments in global health and the need to radically rethink how pharmaceutical systems function to support antiretroviral therapy. Different from many countries that targeted antiretroviral therapy supply systems through largely vertical, donor-driven programs, the Ugandan government and USAID embarked on a broad pharmaceutical system-strengthening focus characterized by: 1) More than 10 years of donor commitment to holistically strengthen interconnected aspects of the pharmaceutical sector, 2) change orientation with innovation and no more business as usual, 3) adherence to USAID’s Collaborating, Learning, and Adapting concept with research support from Harvard University and Makerere University, and 4) the MOH’s *vision* coupled with *trust* among the key implementing stakeholders.

## What are lessons learned?

Among the many lessons learned in implementing the SURE/UHSC program, a few stand out:

- The great need for trusting collaborations and coordination among multiple players within government ministries including the MOH at central, district, and facility levels, religious medical bureaus that oversee the PNFP sector, donors, and their implementing partners.
- The need to maximize use of limited resources by strengthening and harmonizing nation-wide procedures and practices and by making system performance information widely and easily available.
- The need for quality information to enhance management decision making and the importance of building the capacity of health workers and managers at all levels in data use and resource management using standardized procedures and practices.
- The need to design, implement, and monitor performance.
- The need for multi-pronged intervention strategies that target different levels of the system simultaneously, such that operational process changes are backed by policy changes and
- implemention of the program at central level as well as district level to be able to test new interventions and incorporate them into policy.
- The need to assess how well interventions are implemented, how they are perceived, and how well they are working by using the soundest yet feasible longitudinal evaluation methods possible, and to use results to continually adapt and improve system interventions and to pause and modify unsuccessful interventions.

In addition, several of the positive lessons learned came from initially negative experiences that through evaluation and analysis were modified and made successful by applying multipronged strategies to address educational, managerial, regulatory, and financial issues.

In an under-resourced, fragmented health care delivery and financing system such as Uganda’s, a synergy of efforts is needed to optimize health gains from government and donor investments in human and financial resources. This harmonized rather than competitive approach to development aid requires government leadership, time, and a paradigm shift among donors and implementing partners. Building on the impacts of the MOH-SURE/UHSC interventions, the program has evolved as a center of excellence for supporting the supply chain management needs of different implementing partners, and SURE/UHSC staff have supported other partner health programs in their logistic activities. For example, under MOH leadership, the SPARS tool that forms the basis of information in PIP has become the standardized supply management tool for all essential medicines and all managers including donors to the HIV/AIDS programs. SURE/UHSC staff have supported other partner health programs in their logistic activities. USAID’s continuous funding to support the Pharmacy Department’s coordination activities and the commitment of USAID, MOH, and SURE/UHSC staff to foster coordination among implementing have resulted in donor funds used to build on rather than duplicate efforts. In addition, readily available information in the PIP on facility performance (e.g., stock outs, expired medicines) has been crucial for all parties to jointly engage in priority-setting discussions.

## What are visions for the future?

A strong health system is recognized as a prerequisite for reaching several of the United Nations’ sustainable development goals by 2030. Uganda is well-placed to translate continued donor support for health system strengthening into measurable population health and economic benefits; however, the government and donors will need to make critical decisions to maximize limited resources. Moreover, health care and financing needs are continuously changing. What will it take to keep strengthening the Ugandan system while adapting it to address emerging demands?

The SURE/UHSC program has successfully put in place a pharmaceutical system infrastructure on which to build an evolving, learning health system [9]. However, the interconnected components of the pharmaceutical sector strengthening cycle—human resources, information systems, financing and policies and regulations—will require continued, coordinated advances. This will demand political will, deliberate policy and program actions, and innovations to operate increasingly complex pharmaceutical care and financing systems.

### Epidemiological shift to chronic conditions

The rising prevalence of chronic and non-communicable diseases needs both prevention strategies and approaches to making often lifelong treatments accessible, appropriately used, and affordable for the health system and households—progress toward universal health coverage is required to limit unaffordable out-of-pocket expenditures.

If the pharmaceutical and financial systems can adapt to this new epidemiological reality, it will be possible to make innovative and effective yet highly priced treatments for cancers and other chronic diseases available [3].

### Addressing equity

Poverty and income inequality had been on a downward trend until 2012. However, the percentage of Ugandans living under the poverty line increased from 19.7% in 2012/13 to 27.0% in 2016/17 [12–15]. If focused adequately on the poor, moving universal health coverage forward can improve equity in access to quality health care, but major efforts will also be needed to strengthen systems outside of the health sector such as taxation. Without a functioning pharmaceutical system that gives decision makers access to real-time information on who uses medicines, which medicines are used, and how much money households and the government spend on medicines, supplies, and services, universal health coverage efforts in general, and coverage of the poor in particular, will likely fail.

### Community engagement

To make the most of investments in health system strengthening, individuals and households must be empowered to take ownership of the process and to understand their rights to quality health care and access to medicines; in addition, such empowerment can help improve system governance, along with transparency, equity, and accountability. Uganda offers many opportunities to promote stronger linkages between the health system and communities, such as through facilitating uptake of primary prevention, screening, testing (using rapid diagnostics), and early diagnosis and treatment of diverse diseases. Moreover, community feedback can inform health system planning to meet changing population needs. In partnership with United Nations Children’s Fund, United Nations Population Fund, and PATH, Uganda is exploring the feasibility of using standardized tools to enable community feedback on the availability of essential medicines and on health system gaps. Creating a health system that is more responsive to community needs will strengthen national health commodity planning and foster grassroots demand for accountability.

## Summary

To improve Uganda’s health system equitably and sustainably, the country will need to make the most of current resources and it will need more resources. Increased health system investments—from the government or donors—can produce better health only when interconnected pharmaceutical and health care delivery and financing systems function well. The SURE/UHSC program has built a solid foundation on which to base continued holistic pharmaceutical and health system development.

## Abbreviations

EMHS: Essential medicines and health supplies
EMHSLU: Essential Medicines and Health Supplies List of Uganda
FACTS: Financial and commodity tracking system
GDP: Good dispensing practices
GPP: Good pharmaceutical practices
M&E: Monitoring and evaluation
MMS: Medicines management supervisor
MOH: Ministry of Health
PFM: Pharmaceutical financial management
PIP: Pharmaceutical information portal
PNFP: Private not-for-profit
QPPU: Quantification Procurement and Planning Unit
SPARS: Supervision Performance Assessment Recognition Strategy
SURE: Securing Ugandans’ Rights to Essential Medicines [program]
TB: Tuberculosis
TWOS: TB web-based ordering and reporting system
UCG: Uganda Clinical Guidelines
UHSC: Uganda Health Supply Chain [program]
USAID: United States Agency for International Development
VEN: Vital, essential, and necessary
WAOS: Web-based ARV ordering and reporting system
